# Complete Genome Sequences of Two Bovine Alphaherpesvirus 5 Subtype C Strains from Southeast Brazil

**DOI:** 10.1128/mra.01228-21

**Published:** 2022-02-10

**Authors:** Willian P. Paim, Fabrício S. Campos, Samuel P. Cibulski, Camila M. Scheffer, Caroline Tochetto, Ana P. M. Varela, Dennis M. Junqueira, Fabiana Q. Mayer, Phyllis C. Romijn, Edviges M. Pituco, Ana C. Franco, Fernando R. Spilki, Paulo M. Roehe

**Affiliations:** a Laboratório de Virologia, Departamento de Microbiologia, Imunologia e Parasitologia, Instituto de Ciências Básicas da Saúde (ICBS), Universidade Federal do Rio Grande do Sul (UFRGS), Porto Alegre, Rio Grande do Sul, Brazil; b Bioinformatics and Biotechnology Laboratory, Campus de Gurupi, Universidade Federal do Tocantins (UFT), Gurupi, Brazil; c Centro de Biotecnologia (CBiotec), Laboratório de Biotecnologia Celular e Molecular, Universidade Federal da Paraíba (UFPB), João Pessoa, Paraíba, Brazil; d Centro Universitário Ritter dos Reis (UniRitter), Health Science Department, Porto Alegre, Rio Grande do Sul, Brazil; e Centro de Pesquisa em Saúde Animal, Instituto de Pesquisas Veterinárias Desidério Finamor (IPVDF), Departamento de Diagnóstico e Pesquisa Agropecuária, Secretaria de Agricultura, Pecuária e Desenvolvimento Rural, Eldorado do Sul, Rio Grande do Sul, Brazil; f Centro Estadual de Pesquisa em Sanidade Animal Geraldo Manhães Carneiro, Empresa de Pesquisa Agropecuária do Estado do Rio de Janeiro (PESAGRO-RIO), Niterói, Rio de Janeiro, Brazil; g Centro Panamericano de Febre Aftosa, Pedro Leopoldo, Minas Gerais, Brazil; h Laboratório de Microbiologia Molecular, Universidade Feevale, Novo Hamburgo, Rio Grande do Sul, Brazil; DOE Joint Genome Institute

## Abstract

Bovine alphaherpesvirus 5 causes meningoencephalitis in cattle, belongs to the *Herpesviridae* family, and can be divided into subtypes a, b, and c. Limited information is available about subtype c. Here, we report the complete genome sequences of two strains, P160/96, and ISO97/45, isolated from cattle in southeast Brazil.

## ANNOUNCEMENT

Bovine alphaherpesvirus 5 (BoHV-5) is an important agent of meningoencephalitis in cattle, belonging to the family *Herpesviridae,* subfamily *Alphaherpesvirinae,* genus *Varicellovirus* (1), whose genomes are composed by a single double-stranded DNA molecule with 124.8 to 151.6 kbp (https://talk.ictvonline.org/ictv-reports/ictv_online_report/dsdna-viruses/w/herpesviridae/1614/genus-varicellovirus). BoHV-5 is subdivided into subtypes BoHV-5a, BoHV-5b, and BoHV-5c, based on genome restriction endonuclease patterns (2–4). Although BoHV-5 distribution is scarcely known, subtype BoHV-5a seems more widely distributed than BoHV-5b, which has only been detected in Argentina (3–6). Subtype BoHV-5c has only been recovered from a particular region in southeast Brazil (2). There are four complete BoHV-5 genome sequences previously reported, BoHV-5a strain SV507/99 (7) and three BoHV-5b strains (A663, 674/10, and 166/84) (5). Here, two complete genomes of BoHV-5c strains, named P160/96 and ISO97/45, are reported.

**FIG 1:**
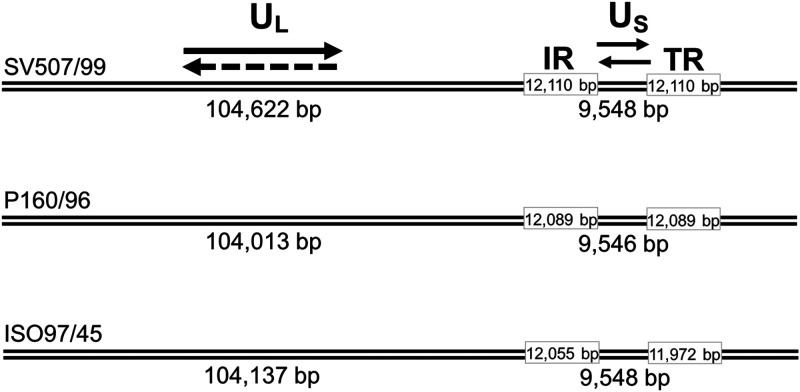
Schematic representation of the genomes reported in this study, highlighting the unique long (UL) and unique short (US) segments and the internal (IR) and terminal (TR) repeat regions. Previously published BoHV-5a strain SV507/99 used as reference (7).

The BoHV-5c strain P160/96 was originally isolated from a case of herpesvirus bovine encephalitis at PESAGRO, in the state of Rio de Janeiro, Brazil. The BoHV-5c strain ISO97/45, also from a case of bovine encephalitis, was recovered from the brain tissues of a calf in 1997. The virus was originally isolated at the Biological Institute of São Paulo, São Paulo, Brazil (8). Both strains had been partially characterized and typed as BoHV-5 by restriction endonuclease and monoclonal antibody analyses (2, 8). For this study, both strains were cultured in Madin-Darby bovine kidney (MDBK) cells (9) and ultracentrifuged (10), and DNA was extracted with phenol-chloroform following standard procedures (11). DNA libraries were prepared with a Nextera kit. High-throughput sequencing was performed in a MiSeq (Illumina) platform, with 500- and 300-cycle kits (version 2) to generate 2 × 250 and 2 × 150 paired-end reads, respectively. The total number of reads mapped to the genomes were 72,887 for P160/96 and 34,096 for ISO97/45. The average read lengths were 250 bp and 150 bp with coverage of 132× and 37×, respectively, of the whole BoHV-5 genome, based on the Lander-Waterman (12) coverage estimate equation. Reads were trimmed using Geneious software (version 9.1) with default settings. Assembly and annotation of the viral genomes were done using template-assisted assembly to the BoHV-5 SV507/99 reference genome (GenBank accession number NC_005261) using a map to reference tools of Geneious version 9.1 with default settings. Both genomes were assembled to full length, including the internal (IR) and terminal repeat (TR) regions (Fig. 1). BoHV-5 genomes showed a classic type D herpesvirus organization (4, 7), with total lengths of 137,741 (P160/96) and 137,712 (ISO97/45) nucleotides (nt), slightly shorter than BoHV-5a SV507/99, which is 138,390 nt long. The difference in genome lengths is shown in Fig. 1. The two BoHV-5 genomes have GC contents of 74.7% (P160/96) and 74.8% (ISO97/45), with 99.4% and 98.9% nucleotide identity to SV507/99, respectively, as determined with the fast Fourier transform (MAFFT) of Geneious version 9.1. The genome sequences of BoHV-5c strains P160/96 and ISO97/45 were also submitted to comparative analyses using MAFFT with default settings, in which each gene was compared individually for identity at the nucleotide level between the different BoHV-5 genomes (Table 1). All bioinformatic tools used here were run with default parameters unless otherwise specified.

**TABLE 1:** Percentages of identity and GC content of nucleotide and amino acid sequences of BoHV-5 strain P160/96 and BoHV-5 strain ISO97/45

		BoHV-5 strain P160/96			BoHV-5 strain ISO97/45		
Gene	Predicted product	% nt identity^*a*^	% aa identity^*b*^	%GC	% nt identity^*a*^	% aa identity^*b*^	%GC
Circ	Myristylated virion protein	99.9	99.6	72.8	99.7	99.6	73
UL54	Regulates and transports RNA	100	100	74.1	99.7	99.8	74.1
UL53	Glycoprotein K	100	100	76.9	99.6	99.7	77
UL52	Component of DNA helicase/primase complex	100	100	76.6	99.4	99.4	76.4
UL51	Palmitoylated protein	99.9	100	75.9	99.7	100	76
UL50	Deoxyuridine triphosphatase	100	100	72.2	99.2	98.8	72.4
UL49.5	Glycoprotein N	100	100	68.8	100	100	68.8
UL49	Tegument protein	100	100	77.2	99.4	99.3	77.2
UL48	*Trans*-inducing factor	99.9	100	72.9	99.5	99.6	73.1
UL47	Tegument phosphoprotein	99.9	100	72.4	99.7	99.9	72.3
UL46	Tegument protein	99.7	99.7	74.8	99.5	99.7	74.6
UL44	Glycoprotein C	99.7	99.8	75.4	99.4	99.4	75.7
UL43	Virion protein	100	100	82	99.1	100	82
UL42	Processivity factor for DNA polymerase	99.9	100	74.5	98.6	98.8	73.8
UL41	Virion host shutoff factor	99.9	100	74	99.5	99.6	73.3
UL40	Ribonucleotide reductase small subunit	100	100	62.8	99.9	100	62.9
UL39	Ribonucleotide reductase large subunit	99.9	99.9	70.6	99.8	99.6	70.4
UL38	Capsid protein	99.8	99.4	75	99.3	99.2	74.4
UL37	Tegument protein	99.9	99.9	79.4	99.5	99.6	79.2
UL36	Very large tegument protein	99.9	99.8	79.8	99.7	99.5	79.8
UL35	Capsid protein	100	100	73.3	100	100	73.3
UL34	Virion protein	100	100	74.8	99.9	100	74.7
UL33	Capsid packaging protein	100	100	71.5	99.7	100	71.2
UL32	Cleavage and packaging protein	99.9	99.8	76.1	99.7	99.8	76.1
UL31	UL34-associated nuclear protein	99.6	98.9	73.6	97	96.6	73.9
UL30	DNA polymerase, catalytic subunit	99.7	99.5	72.2	99.6	99.7	72.5
UL29	Single-stranded DNA binding protein	100	100	73.3	99.7	99.9	73.3
UL28	Cleavage and packaging protein	99.1	98.7	76.2	99.8	99.4	76.5
UL27	Glycoprotein B	100	100	71.6	99.8	99.8	71.4
UL26.5	Capsid scaffolding protein	100	100	79.6	99.7	99.7	79.7
UL26	Capsid maturation serine protease	100	100	78.5	99.7	99.8	78.5
UL25	DNA packaging virion protein	99.8	99.5	77.3	99.6	99.7	77
UL24	Putative membrane-associated protein	98.5	97.5	76.4	99.8	99.6	77.1
UL23	Thymidine kinase	99.9	100	78.6	99.7	100	78.8
UL22	Glycoprotein H	99.9	99.8	76.2	99.5	99.5	76.2
UL21	Tegument protein	99.9	99.8	77.6	99.4	99.7	77.3
UL20	Virion protein	98.6	97.2	76.9	99.9	100	78
UL19	Major capsid protein	99.9	99.9	72.5	99.7	99.9	72.5
UL18	Capsid protein	100	100	75.3	99.8	100	75.4
UL17	Tegument protein	100	100	80.2	99.6	99.7	80.3
UL16	Virion protein	99.8	99.7	77.9	99.9	100	78
UL15	DNA cleavage, packaging protein	99.9	99.7	71.3	99.5	99.9	71.5
UL14	Minor tegument protein	100	100	75.3	99.6	99.1	75.1
UL13	Virion serine/threonine protein kinase	100	100	74.7	99.7	99.6	74.7
UL12	Alkaline exonuclease	98.6	98.8	75.3	99.0	98.8	75.3
UL11	Myristylated protein	100	100	75.2	100	100	75.2
UL10	Glycoprotein M	99.9	100	76	99.4	99.8	75.9
UL9	Origin-binding protein	99.9	99.9	74.5	99.6	99.9	74.5
UL8	Component of DNA helicase/primase complex	100	100	76.7	99.7	99.7	76.6
UL7	Virion-associated protein	100	100	72.7	100	100	72.7
UL6	Virion protein	98.3	98.3	73.4	99.5	99.9	73.8
UL5	Component of DNA helicase/primase complex	99.9	100	66.5	97.0	98.6	68.2
UL4	Nuclear protein	100	100	74.6	99.3	99.5	74.3
UL3.5	Virion protein	100	100	80.1	99.6	99.3	80.1
UL3	Phosphoprotein	99.8	99.5	74.2	97.3	95	74.4
UL2	Uracil DNA glycosylase	100	100	75.5	99.8	99.3	75.3
UL1	Glycoprotein L	100	100	74.4	99.8	100	74.6
UL0.7	Unknown product	99.8	99.5	77.4	NA^*c*^	NA	NA
BICP0	Immediate-early *trans*-activator protein with zinc finger	99.9	100	76.7	99.0	99	76.9
BICP4	Positive and negative gene regulator	100	100	82.2	98.3	97.3	82.2
BICP22	Transcription factor	98.3	98.1	75.3	98.4	97.8	75.1
US1.67	Virion protein	100	100	73.1	99.3	98.8	73
US2	Tegument protein	100	100	72.4	99.0	98.2	72.5
US3	Virion serine/threonine protein kinase	99.9	100	73.9	99.8	99.5	74.1
US4	Glycoprotein G	100	100	68.6	99.8	99.5	68.7
US6	Glycoprotein D	100	100	73.4	99.5	99.5	73.8
US7	Glycoprotein I	99.9	100	76.6	99.6	99.7	76.5
US8	Glycoprotein E	99.9	100	76.3	99.1	98.8	76.2
US9	Virion protein	100	100	76	99.8	99.3	75.8
BICP22	Transcription factor	98.3	98.1	75.3	97.8	96.8	75.2
BICP4	Positive and negative gene regulator	100	100	82.2	98.4	97.5	82

a nt, nucleotide.

b aa, amino acid.

c NA, not applicable.

Unlike other bovine herpesviruses, BoHV-5 has a limited geographical distribution; cases have been commonly reported in South American countries, particularly Brazil and Argentina, and sporadically in other continents (2–9, 13), which makes the origin of BoHV-5 and related outbreaks still a mystery. Previously, one BoHV-5a complete genome sequence (7) and three complete sequences of BoHV-5b were reported (5). Regarding BoHV-5c, formerly called BoHV-5 non-a, non-b (2), no other reports on the occurrence of BoHV-5c infections have been made outside a particular region in southeast Brazil, which seems to comprise the state of Rio de Janeiro, northern São Paulo state, and northeastern Minas Gerais, suggesting that adaptive evolution may have played some role in fixing some of the adaptations that, to date, characterize the BoHV-5c subtype.

However, taxonomy, as currently applied to BoHV-1 and BoHV-5, does not reflect the evolutionary history of these viruses since it is not based on full-genome analyses; REA and monoclonal antibody characterization does not entirely express the complexity of genetic alterations (2, 8). Recently, Romera et al. (5) reported the occurrence of naturally generated interspecific recombinants between BoHV-1 and BoHV-5; obviously, such events can influence type or subtype determination, reinforcing the importance of full-genome analyses to allow for more precise classifications. It is expected that the availability of more complete BoHV-5 genomes, such as strains P160/96 and ISO97/45 reported here, will contribute to a better understanding of the genetic evolution of bovine alphaherpesviruses.

### Data availability.

The genomes have been deposited in NCBI GenBank and are available under accession numbers KY559403 (BoHV-5 strain P160/96) and KY549446 (BoHV-5 strain ISO97/45). The raw sequencing reads were deposited in the NCBI Sequence Read Archive under BioProject accession numbers PRJNA790921 (SRA experiment number SRX13457950 and SRA run ID SRR17280439) and PRJNA790967 (SRA experiment number SRX13458985 and SRA run ID SRR17281500).
